# Visual analysis of research status and hotspots of *Panax notoginseng* from 2000 to 2023: A bibliometric review

**DOI:** 10.1097/MD.0000000000043433

**Published:** 2026-04-24

**Authors:** Kun Lian, Lichong Meng, Xin Li, Lin Li, Junxian Lei, Ji Ouyang, Yuehang Xu, Zhixi Hu

**Affiliations:** a Hunan University of Chinese Medicine, Changsha, China.

**Keywords:** bibliometric analysis, clinical application, *Panax notoginseng*, pharmacologic component, traditional Chinese medicine, visual analysis

## Abstract

**Background:**

*Panax notoginseng* is the most widely used Chinese medicine for preventing and treating ischemic diseases. Because of its good clinical efficacy, high safety, and few adverse reactions, some countries (e.g., the United States, South Korea, Japan, and Australia) have paid it increasing attention, and the number of related studies is also increasing. However, relevant studies are scattered and fragmented, and few systematic summaries and rounded analyses have been conducted. Thus, in this study, we collect and analyze data from the published literature to help researchers understand the research situation in the field and explore research hotspots and frontiers.

**Methods:**

Search and download the literature on *Panax notoginseng* research published from 2000 to 2023 from 3 English databases. We used VOSviewer, Microsoft Excel, and CiteSpace to visually analyze annual publishing trends, publishing countries, institutions, authors, journals, keywords, and references in the field.

**Results:**

A total of 1329 papers were included. The number of papers increased every year, especially in 2022. The leading countries in this field are China, the United States, and South Korea. Kunming University of Science & Technology published the most papers. Most publications were found in the *Journal of Ethnopharmacology*. The most prolific author is Wan Jian-Bo. Keywords with the highest frequency were *Panax notoginseng*, *saponins*, *ginseng*, and *Panax notoginseng saponin*. Eleven clusters and 25 burst keywords were generated.

**Conclusions:**

To date, the research focus in this field has primarily focused on 4 aspects: research on pharmacologic components and quality control, mechanism of action and pathway target research, investigation of applicable diseases and therapeutic effects, and research on cultivation and yield increase. At the same time, research on microscopization, normalization, standardization, and objectification of traditional Chinese medicine is also developing.

## 1. Introduction

*Panax notoginseng* (Burk.) FH Chen, also known as *sanqi*, is among the most widely used herbs in China.^[1]^ Practitioners of traditional Chinese medicine (TCM) believe it has the effect of dispersing stasis, reducing swelling, and relieving pain. Because of its good clinical efficacy, high safety, and few adverse reactions, it is frequently used in the management of heart-related conditions. In addition, it has received increased attention in the United States, South Korea, Japan, Australia, and other countries.^[2]^ *P notoginseng* can be used for a variety of diseases, such as prostate cancer,^[3]^ diabetic retinopathy,^[4]^ acute pancreatitis,^[5]^ depression, and osteoarthritis.^[6,7]^ Previous researchers have found that *P notoginseng* includes many pharmacologic components such as *P notoginseng* saponins (PNS), quercetin, kaempferol, and *P notoginseng* polysaccharide.^[8]^ It also has many pharmacological effects, such as activation of blood circulation, hemostasis, replenishing blood, analgesia, anti-inflammatory, antitumor, antioxidation, immune regulation, and neuroprotection effects.^[9–12]^Over the previous 20 years, a large number of research studies on *P notoginseng* have been published, including animal experiments,^[13]^ cell experiments,^[14]^ clinical trials,^[15]^ cultivation studies,^[16]^ theoretical discussions, and reviews.^[17]^ However, the relevant studies are scattered and fragmented, and few systematic summaries and comprehensive analyses have been conducted. Therefore, with the help of bibliometrics and visual analysis technology, in this study, we collect and analyze data from the published literature to help researchers understand the research status in this field and explore research frontiers and hot spots.

Statistical Bibliography, the previous name for bibliometrics, was first used in 1969. This is a study of academic publishing that uses statistics to describe publishing trends and highlights the relationships among published works.^[18]^ The research shows that visualization software such as CiteSpace can objectively and fully reflect the development of related fields.^[19]^

In this study, we analyzed publications on *Panax notoginseng* using a bibliometric approach and systematically evaluated the status of recent *Panax notoginseng*, current research priorities, and new research trends for systematic evaluation, highlighting landmark results and pointing out future research directions. At the same time, we hope to provide reference and guidance for future clinical and scientific research.

## 2. Methods

### 2.1. Search strategies

We searched the literature on *P notoginseng* using the keywords *Panax notoginseng*, *sanqi*, *sanqi*, and *pseudo-ginseng* in the Web of Science Core Collection, Medline Complete, and PubMed databases, and the time range was from 2000 to 2023. Table 1 shows the detailed search formula. The retrieved literature was exported in “full records and cited literature” format.^[20]^

**Table 1:** Databases and search formulas.

Database	Search formula
Web of Science Core Collection	((((TS = (*Panax notoginseng*)) OR TS = (san qi)) OR TS = (sanqi)) OR TS = (pseudo-ginseng)) OR TS = (notoginseng radix)
Medline complete	SU = *Panax notoginseng* OR san qi OR sanqi OR pseudo-ginseng OR notoginseng radix
Pubmed	((((*Panax notoginseng*[Title/Abstract]) OR (san qi[Title/Abstract])) OR (sanqi[Title/Abstract])) OR (pseudo-ginseng[Title/Abstract])) OR (notoginseng radix[Title/Abstract])

### 2.2. Data inclusion and exclusion criteria

Inclusion criteria included articles and review articles on notoginseng research, and the full text is published in English. Exclusion criteria consisted of the following: literature not related to the subject of this study; letters, notes, and corrections; studies with incomplete or unidentifiable information, such as title, abstract, keywords, and author; systematic reviews and meta-analyses; repeated publications; retracted articles.

### 2.3. Data standardization

According to the inclusion and exclusion criteria, the final included literature was obtained. In this study, we excluded systematic reviews and meta-analyses. On the 1 hand, such studies belong to the reanalysis of the original research. This study included the original research and may overlap with systematic reviews and meta-analyses. On the other hand, this article also belongs to reviews in a broad sense and should not be included in systematic reviews and meta-analyses any longer.

According to the relevant standards of TCM terms, combine the keywords with the same meaning. The name of the institution is processed based on the information displayed on its official website. Authors with the same name and surname make a comprehensive judgment based on information such as their affiliated institution and research content.

### 2.4. Data analysis and mapping

We exported the final included documents and named the file “download.” We used CiteSpace 6.2.R2 software (Professor Chaomei Chen, Drexel University, Philadelphia) for complete data conversion and drawing the visualization map. We set the parameters as follows: time slicing = January 2000 to June 2023, selection criteria = top 50, and years per slice = 2. For pruning, we used Pruning Tender Networks, Pathfinder, with the remainders of the sections left as default. Based on the purpose of this research, we selected “country, institution, keyword” for the node type area. We used keyword select burst detection from the “Keyword Analysis” submenu. The importance of nodes in a network was measured by centrality, which was a parameter. The higher the centrality of a node, the more frequently it co-occurred with other nodes, and the more important it was in the entire network.^[21,22]^

VOSviewer 1.6.18.0 Software parameter Settings: The number of journal publications ≥ 5, the number of citations of journals ≥ 80, the number of author publications ≥ 5, the number of citations of authors ≥ 20, the number of citations of references ≥ 20, and the rest keep the default values.

We saved the CiteSpace and VOSviewer running results to Microsoft Office Excel 2019 for data statistics and tabulation. Images were generated using Bioinformatics (www.bioinformatics.com.cn) and Bibliometrics (bibliometric.com).

## 3. Results

### 3.1. General information and annual publication output

**Figure 1.**
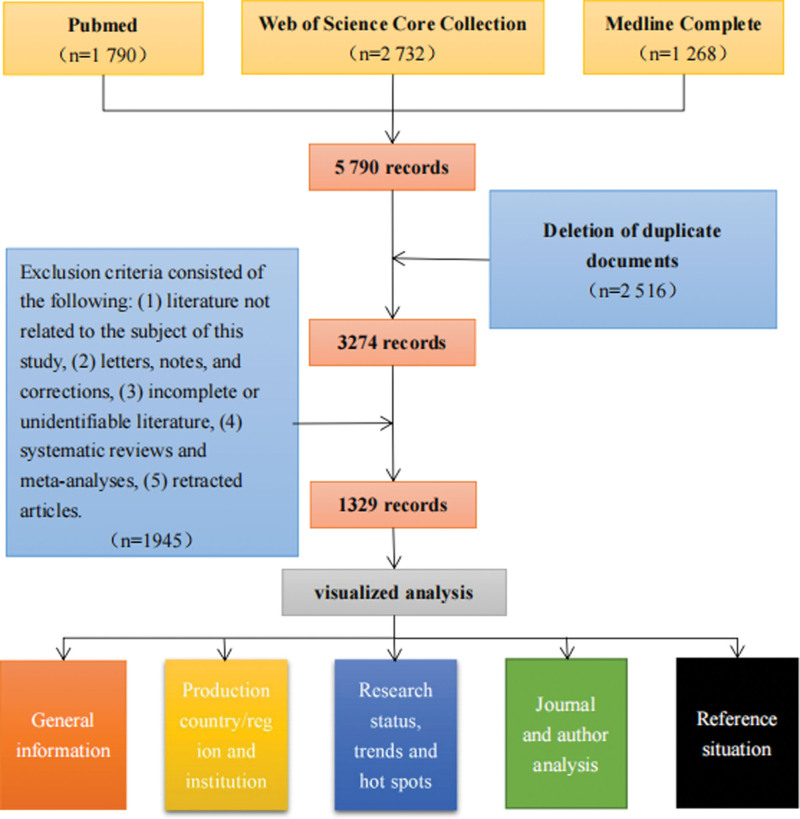
Flowchart of literature selection.

Since the beginning of the 21st century, a total of 5790 articles have been collected in the 3 databases. After applying the inclusion and exclusion criteria, we finally included 1329 articles. Figure 1 shows the flowchart of literature selection and processing.

**Figure 2.**
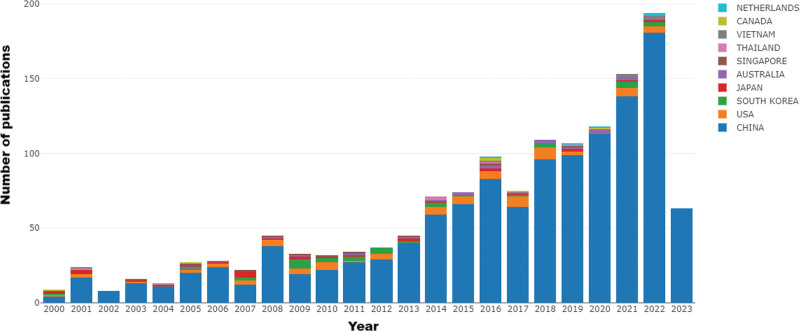
Annual number of publications.

The number of papers published each year is an important indicator of the development trend of the field and, to a certain extent, reflects the progress of knowledge in the discipline.^[23,24]^ The annual number of publications in this field over the past 24 years is visually displayed in the form of a histogram, as shown in Figure 2. The number of publications increased from 2000 to 2023, with China serving as the main country of authorship. The trend in the published literature in this field can be broadly divided into 2 stages. In the first period, from 2000 to 2013, the trend was flat but increased slightly. In the second stage, from 2014 to 2023, there was a clear growth trend in the number of articles published. The year with the highest number of published articles was 2022, with >150. From the overall trend, we determined that the related research on *P notoginseng* is developing and receiving increasing attention, and it has entered a rapid development stage, especially in the most recent 10 years.

### 3.2. Distribution of countries or territories

**Figure 3.**
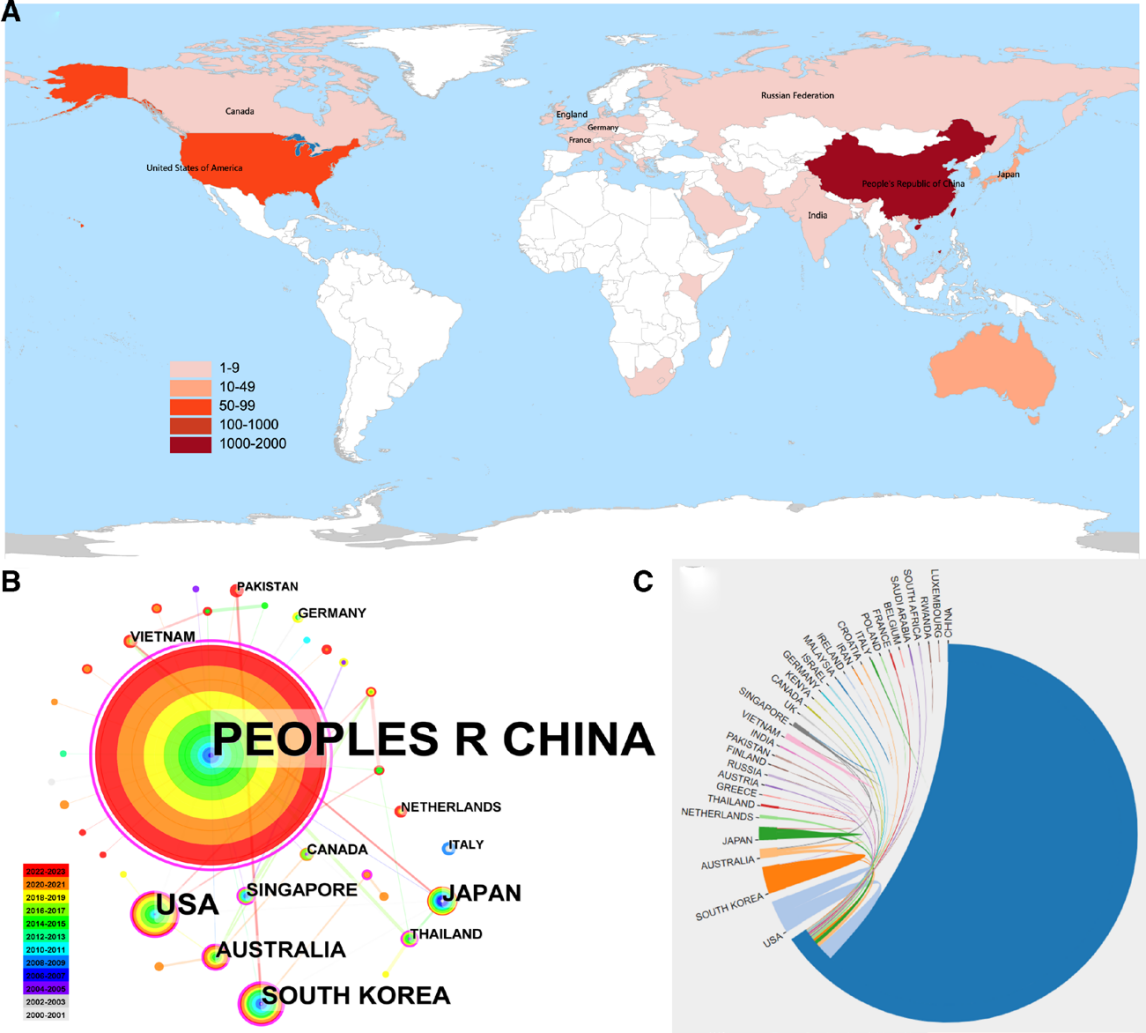
(A) Distribution of *Panax notoginseng* studies worldwide. (B) Map of cooperation of countries/territories. (C) Countries/territories collaboration map.

Collaboration maps can show the collaboration between countries or regions in the research field, so as to provide a reference for evaluating the academic influence of a country or region.^[25,26]^ A total of 35 countries/territories have contributed to the research of *P notoginseng*, and the number of publications issued by each country/territory is shown in Figure 3A. Six countries/territories have published >10 papers, namely, People’s Republic of China (n = 1231), United States (n = 71), South Korea (n = 36), Japan (n = 26), Australia (n = 21), and Singapore (n = 12). Table 2 shows the top 10 countries.

**Table 2:** Top 10 productive countries/territories related to research.

Rank	Country	Year	Publications (%)	Centrality
1	Peoples R China	2000	1231 (92.6)	1.59
2	USA	2000	71 (5.3)	0.15
3	South Korea	2000	36 (2.7)	0.22
4	Japan	2000	26 (2.0)	0.07
5	Australia	2005	21 (1.6)	0.16
6	Singapore	2003	12 (0.9)	0.13
7	Canada	2000	6 (0.5)	0
8	Vietnam	2001	6 (0.5)	0.01
9	Thailand	2001	6 (0.5)	0.14
10	Germany	2003	5 (0.4)	0

Figures 3B and 3C show the partnerships and research time between different countries. The research in this field was first carried out in countries/territories such as the People’s Republic of China (Peoples R China), the United States, South Korea, Japan, and Canada in 2000, and related research began in Australia in 2005. The highest centrality ranking was noted in Peoples R China (1.59), followed by South Korea (0.22), Australia (0.16), and the United States (0.15).

### 3.3. Distribution of institutions

**Figure 4.**
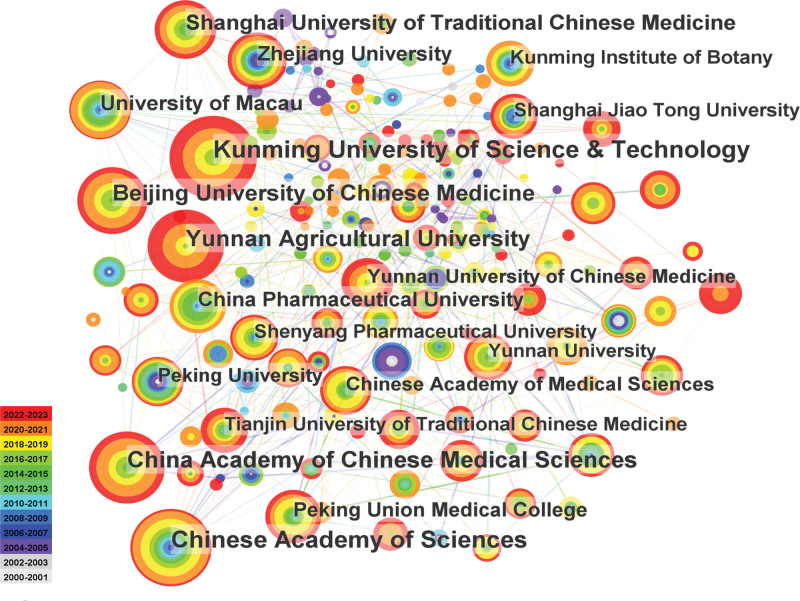
Map of institutional partnerships.

As shown in Figure 4, a total of 969 institutions have conducted research in this field, of which 103 institutions have published >5 papers each. Kunming University of Science & Technology (n = 101) published the most papers, followed by Chinese Academy of Sciences (n = 91), China Academy of Chinese Medical Sciences (n = 76), Yunnan Agricultural University (n = 73), and Beijing University of Chinese Medicine (n = 66). The University of Macau has the highest centrality (0.23), followed by the Chinese Academy of Sciences (0.16), the Chinese University of Hong Kong (0.16), Shanghai University of TCM (0.14), and Shanghai Jiao Tong University (0.13). Table 3 shows the top 10 institutions in terms of publication volume.

**Table 3:** Top 10 institutions by publications.

Rank	Institutions	Y	Publications (%)	Centrality
1	Kunming University of Science & Technology	2010	101 (7.6)	0.11
2	Chinese Academy of Sciences	2001	91 (6.8)	0.16
3	China Academy of Chinese Medical Sciences	2006	76 (5.7)	0.08
4	Yunnan Agricultural University	2008	73 (5.5)	0.03
5	Beijing University of Chinese Medicine	2010	66 (5.0)	0.05
6	Shanghai University of Traditional Chinese Medicine	2008	58 (4.4)	0.14
7	University of Macau	2006	50 (3.8)	0.23
8	Zhejiang University	2003	48 (3.6)	0.04
9	China Pharmaceutical University	2003	44 (3.3)	0.08
10	Peking Union Medical College	2005	42 (3.2)	0

### 3.4. Productive journals and co-cited journals

**Figure 5.**
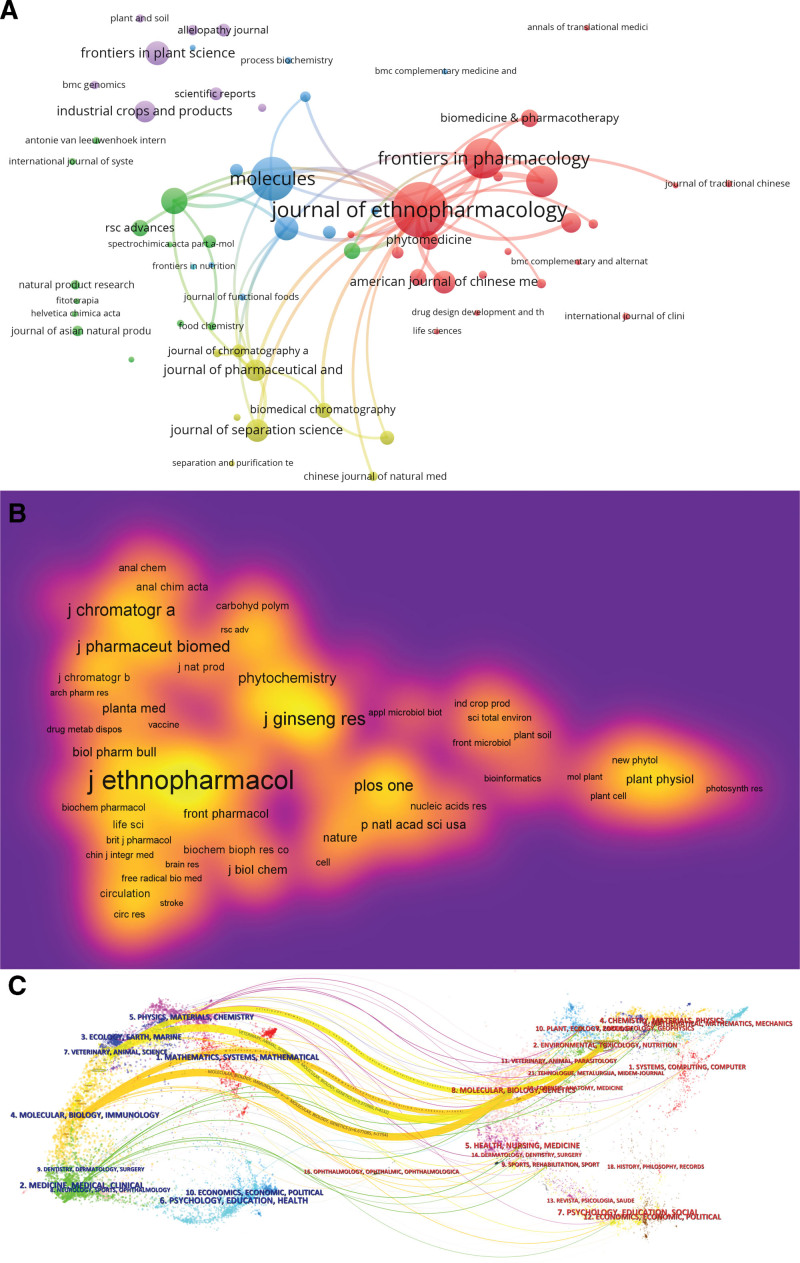
(A) Map of journal publications. (B) Map of the density of co-cited journals. (C) Dual-map overlay of journals.

To determine the journals with the most publications and co-citations related to *P notoginseng*, we analyzed the journals and co-cited journals. The results showed that among 459 journals related to *P notoginseng* research literature, 65 journals published >5 articles: *Journal of Ethnopharmacology* (n = 50), followed by *Molecules* (n = 39), *Frontiers in Pharmacology* (n = 36), *Journal of Ethnopharmacology* (n = 50), *Evidence-Based Complementary and Alternative Medicine* (n = 28), and *Journal of Ginseng Research* (n = 23). Figure 5A shows the published journals.

A total of 6686 journals were cited, of which 185 were cited >50 times and 108 were cited >80 times. Figure 5B shows the density of the co-cited journals. The most frequently co-cited journal is the *Journal of Ethnopharmacology* (n = 1289), followed by *Journal of Ginseng Research* (n = 668), *Journal of Agricultural and Food Chemistry* (n = 644), and *Journal of Chromatography A* (n = 593). Table 4 shows the top 10 journals by number of publications or citations.

**Table 4:** The top 10 journals and co-cited journals related to *Panax notoginseng*.

Rank	Journal	Publications (%)	IF/JCR (2022)	Co-cited journal	Citations	IF/JCR (2022)
1	Journal of Ethnopharmacology	50 (3.8)	5.195/Q1	Journal of Ethnopharmacology	1289	5.195/Q1
2	Molecules	39 (2.9)	4.927/Q2	Journal of Ginseng Research	668	5.735/Q1
3	Frontiers in Pharmacology	36 (2.7)	5.988/Q1	Journal of Agricultural and Food Chemistry	644	5.895/Q1
4	Evidence-based Complementary and Alternative Medicine	28 (2.1)	2.650/Q3	Journal of Chromatography A	593	4.601/Q1
5	Journal of Ginseng Research	23 (1.7)	5.735/Q1	Journal of Pharmaceutical and Biomedical Analysis	566	3.571/Q2
6	Frontiers in Plant Science	21 (1.6)	6.627/Q1	Plos One	518	3.752/Q2
7	Journal of Agricultural and Food Chemistry	21 (1.6)	5.895/Q1	Chemical & Pharmaceutical Bulletin	504	1.903/Q3
8	American Journal of Chinese Medicine	20 (1.5)	6.005/Q1	Phytochemistry	450	4.004/Q1
9	Journal of Separation Science	20 (1.5)	3.614/Q2	Molecules	448	4.927/Q2
10	Industrial Crops and Products	19 (1.4)	6.449/Q1	Food Chemistry	428	9.231/Q1

Data obtained from https://www.iikx.com.

Figure 5C displays a dual-map overlay of journals. The study’s cited journal subject areas are located on the left side of the graph, whereas the cited journal subject areas are located on the right side. The curve in the figure illustrates the trajectory of literature in the field of *P notoginseng* research across disciplines. As can be seen in the figure, there are 4 main reference tracks: molecular/biology/immunology to molecular/biology/genetics and environmental/toxicology/nutrition, the veterinary/animal/science to molecular/biology/genetics and environmental/toxicology/nutrition.

### 3.5. Authors and cited authors analysis

**Figure 6.**
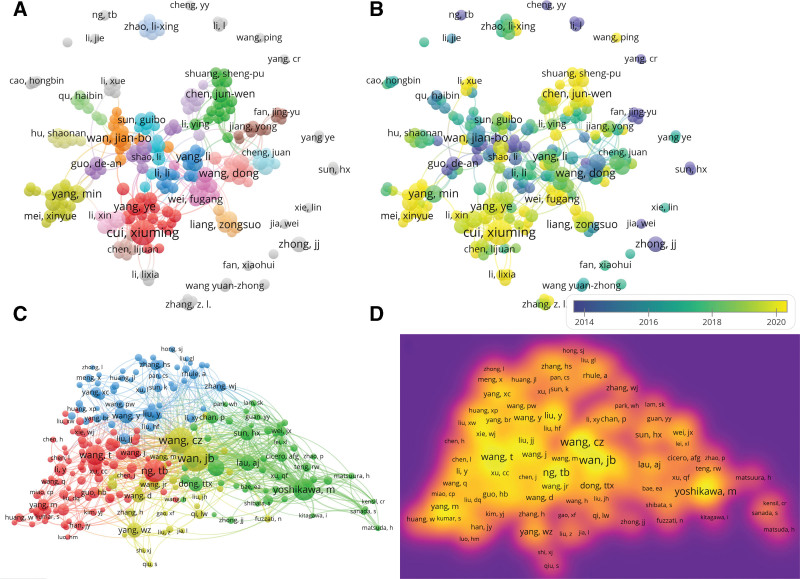
(A) Map of the authors. (B) Time series diagram of published authors. (C) Map of co-cited authors. (D) Map of the density of co-cited authors.

The final literature included studies published by 5851 authors, of whom 216 published >5 articles and 49 published >10 articles. Table 5 lists the 10 authors who published the most papers. It can be seen that Cui Xiu-ming published the most articles with a total of 57, followed by Wan Jian-Bo (n = 27), Wang Dong (n = 22), Yang Min (n = 22), He Xia-hong (n = 21), and Yang Ye (n = 21). According to the network of authors participating in the study on *P notoginseng* (Fig. 6A), there are more small teams of researchers, but less interteam cooperation, and the degree of centrality of the researchers is small. Figure 6B presents a researcher’s time series diagram that shows the situation of researchers in different periods. Yellow in the diagram represents recent researchers, such as Cui Xiu-ming, Chen Jun-wen, Mei Xin-yue, and Shuang Sheng-pu.

**Table 5:** Top 10 authors by publication or cited authors by count.

Rank	Author	Publications (%)	Centrality	Cited author	Citations	Centrality
1	Cui Xiu-ming	57 (4.3)	0.04	Wan JB	265	0.16
2	Wan Jian-Bo	27 (2.0)	0.03	Wang CZ	263	0.14
3	Wang Dong	22 (1.7)	0.01	Ng TB	213	0.06
4	Yang Min	22 (1.7)	0	Yoshikawa M	200	0.13
5	He Xia-hong	21 (1.6)	0.03	Wang T	173	0.06
6	Yang Ye	21 (1.6)	0.05	Li L	127	0.05
7	Liu Di-qiu	20 (1.5)	0.01	Lau AJ	116	0.15
8	Yang Chong-Ren	19 (1.4)	0	Yang WZ	108	0.05
9	Zhang Ying-Jun	18 (1.4)	0	Dong TTX	106	0.07
10	Chen Jun-Wen	18 (1.4)	0.01	Liu Y	104	0.03

Co-cited authors refer to the phenomenon in which 2 or more authors are simultaneously cited by other literature. As shown in Figure 6C, there are 25,097 co-cited authors in this study, who can be roughly divided into 4 categories. There were 239 authors cited >20 times and 137 authors cited >30 times. In Figure 6D, a brighter yellow indicates a greater number of citations. The author with the highest number of co-citations was Wan J. B. (n = 265), followed by Wang C. Z. (n = 263), Ng T. B. (n = 213), and Yoshikawa M. (n = 200). Table 5 lists the top 10 authors with the highest total number of citations.

### 3.6. Analysis of keywords

#### 3.6.1. Co-occurrence of keywords analysis

Keywords represent the core of the paper but also provide a high-level generalization of the paper topic.^[27,28]^ Therefore, analyzing keywords of the paper can help us understand its theme and core and determine the research hot spots and frontiers in this field.^[29]^ After replacing synonyms or similar words, we obtained a keyword co-occurrence graph.

**Figure 7.**
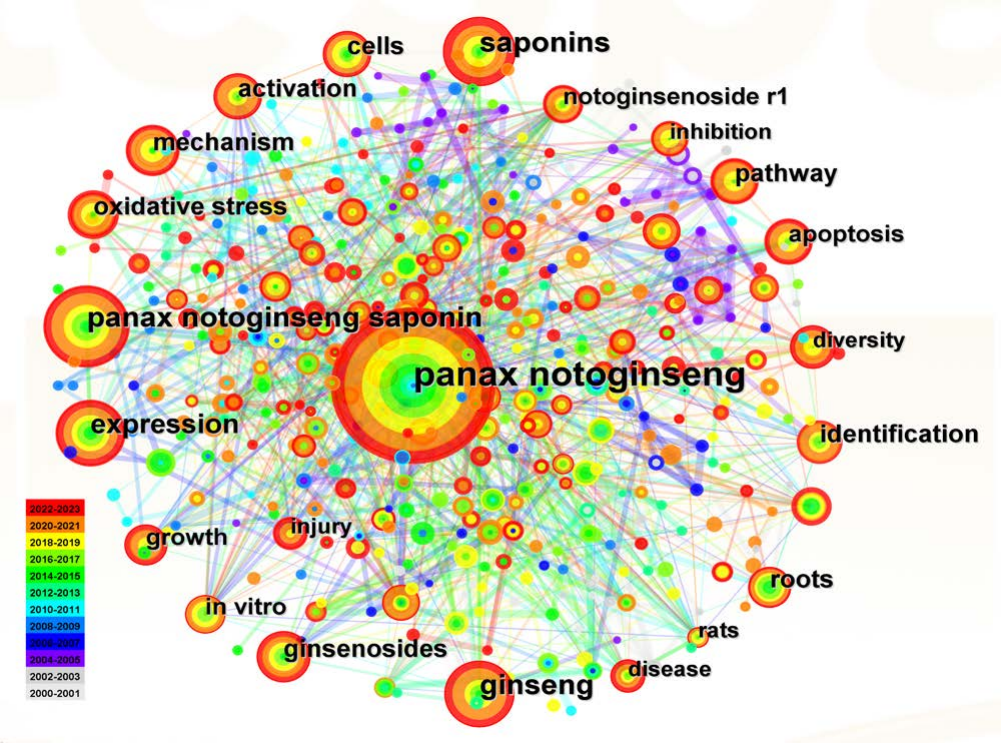
Map of keyword co-occurrence.

Figure 7 shows that there are 467 keywords and 1494 lines, and the density value is 0.0137. Each node represents a keyword, and the larger the circle, the higher the frequency of the keyword. Each circle is composed of a number of concentric color rings; the color of the ring represents the time of paper publication containing relevant keywords, and the width of the color ring represents the number of papers published over time. The thickness of the line indicates how often 2 keywords appear together. The keywords with the highest frequency include *Panax notoginseng* (n = 581), saponins (n = 171), *Panax notoginseng* saponin (n = 167), and expression (n = 132). Keywords with high centricity were extraction (0.12), *Panax notoginseng* (0.11), in vitro (0.1), growth (0.1), performance liquid chromatography (0.1), extract (0.1), and constituents (0.1). The top 20 keywords with the highest frequency are sorted in Table 6.

**Table 6:** The top 20 keywords associated with the *Panax notoginseng*.

Rank	Keywords	Count	Centrality	Rank	Keywords	Count	Centrality
1	*Panax notoginseng*	581	0.11	11	Oxidative stress	75	0.02
2	Saponins	171	0.09	12	Pathway	72	0.04
3	Ginseng	167	0.06	13	Identification	71	0.07
4	*Panax notoginseng* saponin	167	0.05	14	Apoptosis	69	0.04
5	Expression	132	0.03	15	In vitro	68	0.1
6	Ginsenosides	90	0.04	16	Growth	62	0.1
7	Roots	86	0.04	17	Notoginsenoside r1	57	0.06
8	Activation	85	0.04	18	Inhibition	51	0.03
9	Cells	85	0.05	19	Disease	48	0.03
10	Mechanism	77	0.02	20	Rats	45	0.09

#### 3.6.2. Keyword cluster analysis

**Figure 8.**
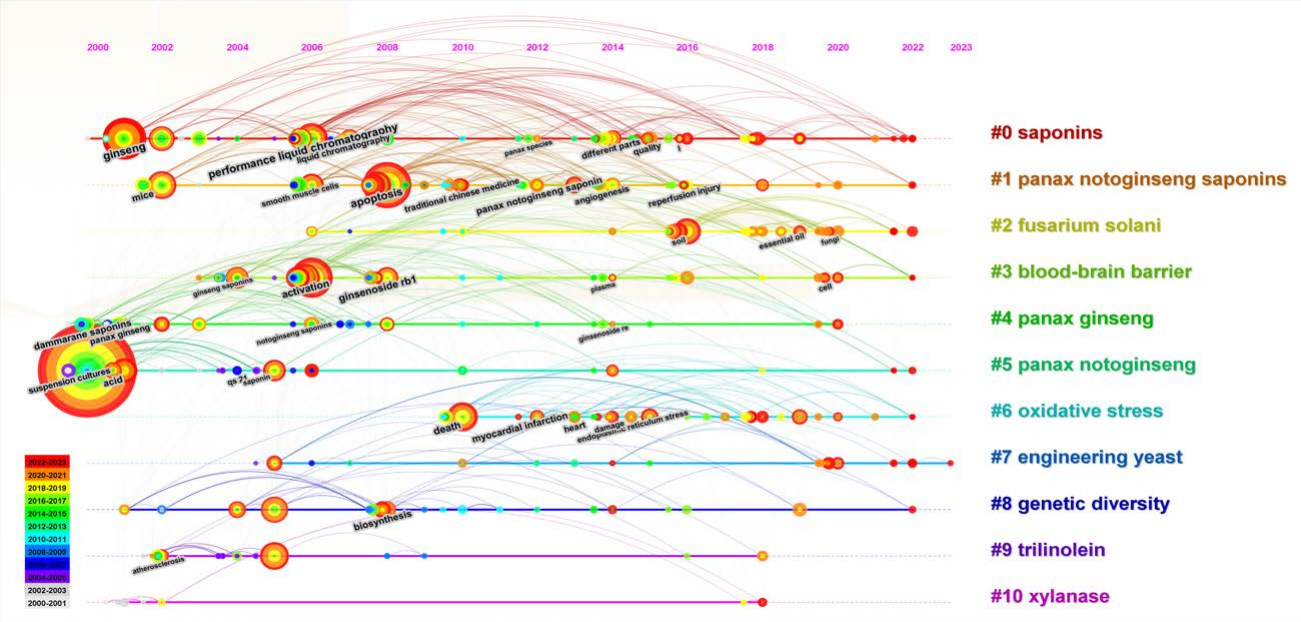
Timeline view of keywords.

Figure 8 shows a keyword time graph, which displays the evolution of high-frequency keywords in each cluster. In addition, the graph can also help us determine the evolution trajectory of research periods and research directions in specific topics. Combined with the clustering data in Table 7, we found that there are roughly 11 research directions in this field.

**Table 7:** Keyword clustering data sheet.

Cluster ID	Size	Silhouette	Mean (Y)	Label (LLR)
#0	76	0.79	2011	Saponins, pressurized liquid extraction, *Panax notoginseng* leaves
#1	62	0.7	2011	*Panax notoginseng* saponins, notoginsenoside r1, inflammation
#2	52	0.715	2017	Fusarium solani, microbial diversity, antifungal activity
#3	48	0.728	2010	Blood-brain barrier, pharmacokinetics, excretion
#4	39	0.778	2006	Panax ginseng, bioactive saponins, ginsenoside rg1
#5	39	0.863	2005	*Panax notoginseng*, hemolysis, saponin content
#6	38	0.759	2015	Oxidative stress, mitochondria, mitophagy
#7	30	0.785	2017	Engineering yeast, glycosyltransferase genes, polysaccharide
#8	28	0.89	2009	Genetic diversity, gene expression, cultivar breeding
#9	18	0.862	2005	Trilinolein, cardiomyocyte hypertrophy, reactive oxygen species
#10	11	0.952	2005	Xylanase, subcellular distribution, acid soil

The earliest and most persistent are #0 Saponins and #5 *Panax notoginseng*, which appeared in 2000 and lasted for 22 years until 2022. Also lasting >20 years are #1 *Panax notoginseng* saponins, #4 panax ginseng, and #8 genetic diversity. The latest and shortest-lasting is #6 oxidative stress, which appeared from 2010 to 2022, lasting a total of 12 years. The #7 engineering yeast, which will last until 2023, has certain research prospects.

#### 3.6.3. Burst keywords analysis

**Figure 9.**
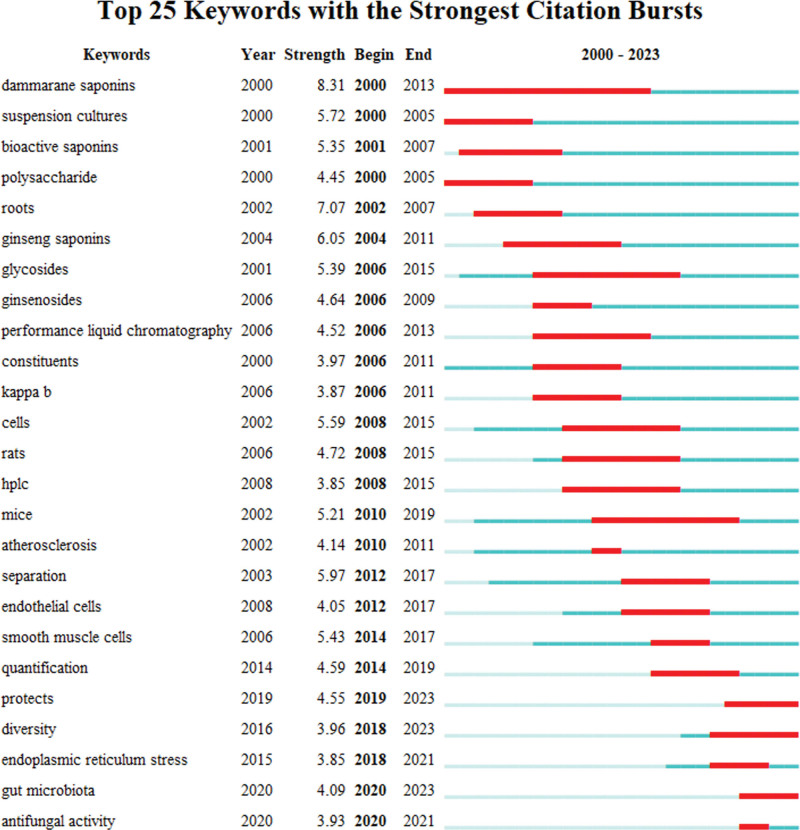
Top 25 keywords with the strongest citation bursts.

Burst detection is an algorithm to determine the change in a variable over a certain period of time. Based on the length of the research content, the burst detection of keywords can forecast future research directions and reflect research trends at different stages.^[28,30]^ The intensity value is an index that measures the intensity of the citation outbreak, and the greater the intensity value, the stronger the outbreak. Figure 9 shows the top 25 keywords for outbreak value. The duration of the outbreak ranged from 2 to 14 years, and the intensity ranged from 3.85 to 8.31 years. Dammarane saponins had the highest intensity (8.31) and the longest duration (n = 14). The keywords such as diversity and gut microbiota that persist today indicate that they are still a focus of future research.

### 3.7. Analysis of references

**Figure 10.**
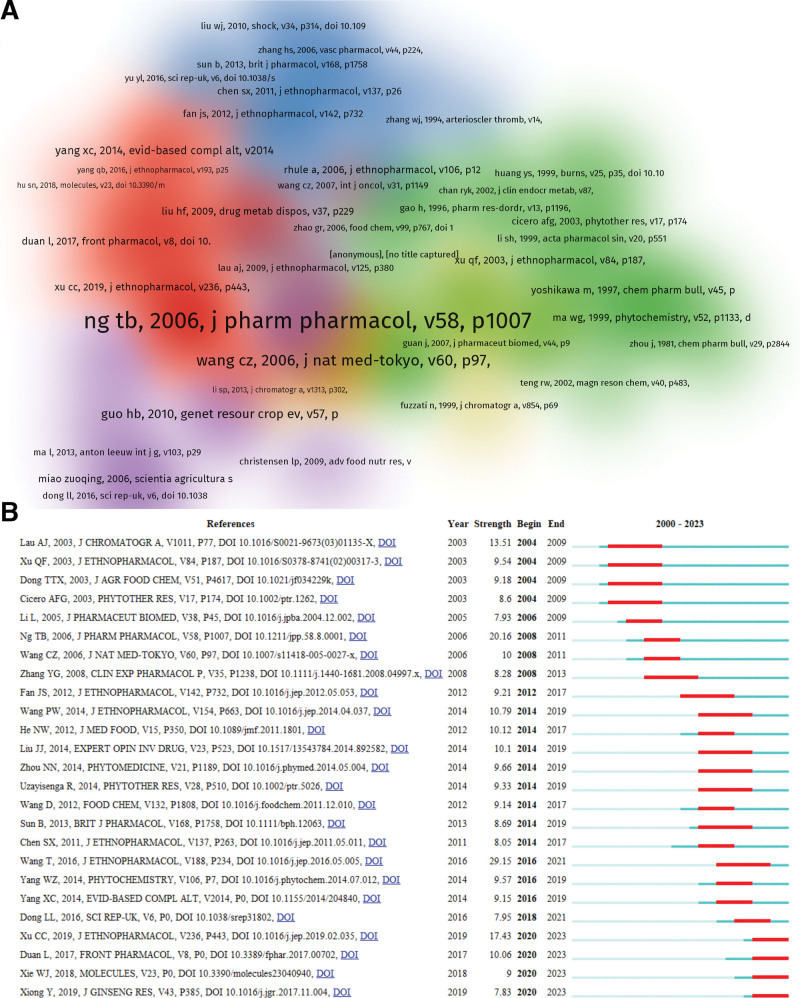
(A) Map of co-cited references. (B) Top 25 co-cited references with the strongest citation bursts.

Figure 10A shows the map of co-cited references. The most frequently cited paper is the paper published by Ng T. B. in the *Journal of Pharmacy and Pharmacology* in 2006. The 10 most cited articles are sorted as shown in Table 8. Citation bursts are references that are frequently cited over a long period of time. Figure 10B shows the top 25 most cited references. In the results of explosion intensity, Wang T.^[32]^ had the highest explosion intensity, which was 29.15. In addition, Xu C. C.,^[41]^ Duan L.,^[42]^ Xie W. J.,^[43]^ and Xiong Y.^[44]^ received more attention.

**Table 8:** Top 10 most cited publications.

Rank	Title	First author	Journal	Y	Citation	Centrality	References
1	Pharmacological activity of sanchi ginseng (*Panax notoginseng*)	Ng TB	J Pharm Pharmacol	2006	195	0.11	^[31]^
2	Traditional uses, botany, phytochemistry, pharmacology and toxicology of *Panax notoginseng* (Burk.) FH Chen: a review	Wang T	J Ethnopharmacol	2016	147	0.05	^[32]^
3	Phytochemical and analytical studies of *Panax notoginseng* (Burk.) FH Chen	Wang CZ	J Nat Med-Tokyo	2006	112	0.12	^[33]^
4	Chemical assessment of roots of *Panax notoginseng* in China: regional and seasonal variations in its active constituents	Dong TTX	J Agr Food Chem	2003	98	0	^[34]^
5	Chemical characteristics for different parts of *Panax notoginseng* using pressurized liquid extraction and HPLC-ELSD	Wan JB	J Pharmaceut Biomed	2006	75	0.1	^[35]^
6	Sanchi ginseng (*Panax notoginseng* (Burkill) F. H. Chen) in China: distribution, cultivation and variations	Guo HB	Genet Resour Crop Ev	2010	71	0.11	^[36]^
7	Analysis of saponins in raw and steamed *Panax notoginseng* using high-performance liquid chromatography with diode array detection	Lau AJ	J Chromatogr A	2003	57	0.05	^[37]^
8	Protective effects of *Panax Notoginseng* Saponins on cardiovascular diseases: a comprehensive overview of experimental studies	Yang XC	Evid-based Compl Alt	2014	57	0.01	^[38]^
9	Effects of steaming the root of *Panax notoginseng* on chemical composition and anticancer activities	Sun S	Food Chem	2010	56	0	^[39]^
10	Analysis of relative gene expression data using real-time quantitative PCR and the 2 (T)(-Delta Delta C) method	Livak KJ	Methods	2001	51	0	^[40]^

## 4. Discussion

### 4.1. Hotspots and frontiers

Due to the limitations of factors such as region and discipline, the cooperation among authors is not close enough, and the scale of cooperation is relatively small. In the future, it is suggested that scholars actively organize and carry out in-depth exchanges across regions and disciplines to promote the development of related research and fields of the TCM *Panax notoginseng*. Through our systematic and comprehensive analysis, we found that researchers in this field are mainly concerned about the following 4 aspects.

1. Research on pharmacologic components and quality control

It is well known that Chinese medicine is developing toward objectification, standardization, and microcosm. Keywords in this direction include *Panax notoginseng* saponin, saponins, notoginsenoside R1, ginsenoside Rg1, ginsenoside Rb1, polysaccharide, performance liquid chromatography, and quality control. The analysis of pharmacologic components can help clarify the mechanism and target of drug action, and help drug identification and quality control. Modern pharmacologic studies have found that *P notoginseng* saponin mainly contains various saponins and polysaccharides, such as *P notoginsenoside* R1 (NG-R1), ginsenoside Rg1, and ginsenoside Rb1. Du Y. Q.^[45]^ believed that notoginseng saponins were the main active components in notoginseng leaves, which could reduce inflammation and promote fibroblast proliferation and angiogenesis. They can also be combined with bone mesenchymal stem cells to treat diabetic skin ulcers. Wang M. J.^[46]^ stated that *P notoginseng* saponin R1 was one of the main components of *P notoginseng* and had neuroprotective effects. It was also found that NGR1 can reduce the levels of tumor necrosis factor-α (TNF-α), interleukin (IL)-6, and IL-1β in the hippocampus of rats induced by isoflurane, promote the expression of miR-29a, and inhibit the inflammatory response, so as to alleviate the nerve function injury induced by isoflurane. Only by ensuring the quality of the drug can the therapeutic effect be guaranteed. At present, research hotspots include establishing authoritative, scientific, and guiding TCM quality evaluation standards with TCM characteristics and drug use characteristics, as well as the mode transformation from single-component determination to multicomponent detection and overall control. Some techniques have also been applied to the field, such as performance liquid chromatography,^[47]^ front-face synchronous fluorescence spectroscopy,^[48]^ and low- and high-field nuclear magnetic resonance.^[49]^

1. Study on the mechanism of action and pathway targets

Studying the mechanism of action is helpful for clarifying the effect of drug action and is conducive to precise treatment and targeted drug use. Typical keywords include oxidative stress, apoptosis, inflammation, gut microbiota, pathway, endoplasmic reticulum stress, blood-brain barrier, and mitophagy. At present, this direction is mainly realized through network pharmacology, animal models, and cell experiments. Li D. D.^[50]^ found that *Panax notoginseng* stem could inhibit apoptosis and inflammation in rats with renal ischemia-reperfusion injury, and reduce the area of renal tubule cell injury and renal infarction area. Lichota A.^[51]^ reported that NG-R1 could protect intestinal microbiota by changing the REDOX state. Wang L.^[52]^ found that PNS could reduce the proinflammatory cytokines IL-6, IL-1β, IL-17, and TNF-α; reduce *Bacteroides* spp.; and increase the abundance of *Akkermansia* spp., thereby regulating gut flora and treating inflammatory bowel disease. Xiao Q.^[53]^ found that PNS can inhibit the activation of the NOD-like receptor thermal protein domain-associated protein 3 inflammasome, promote mitochondrial autophagy through the PINK1/Parkin pathway, and alleviate cerebral ischemia-reperfusion injury in rats. In sum, TCM has the characteristics of multiple targets and a complex mechanism of action; as such, clarifying its mechanism and pathway of action is a hot research topic.

1. Study on applicable diseases and symptoms and the curative effect

The key to Chinese medicine is the curative effect. In the final analysis, research on notoginseng should be applied clinically and used to relieve human pain. Notoginseng has the effect of dispersing stasis, stopping bleeding, and relieving swelling and pain. Adaptive disorders include atherosclerosis, ischemia-reperfusion injury, stroke, Alzheimer’s disease, myocardial infarction, and cerebral ischemia. However, few clinical studies in this field have been conducted, mainly via cell and animal experiments, network pharmacology, and molecular docking. Zeng J. J.^[54]^ proposed that NG-R1, as a new saponin isolated from notoginseng, can inhibit JNK/p38 signal transduction, reduce myocardial infarction size, and alleviate myocardial apoptosis and myocardial injury by inhibiting the activity of transforming growth factor-β-activated protein kinase 1. Ultimately, it alleviates myocardial ischemia-reperfusion damage. Shao R. F.^[55]^ proposed that PNS inhibited the NF-κB signaling pathway; inhibited the expression of NF-κB p65, TNF-α, IL-6, and IL-1β in mouse aortic root tissue; decreased the levels of triglycerides, low-density lipoprotein cholesterol, and total cholesterol; and increased the levels of high-density lipoprotein cholesterol. It was also found to reduce lipid deposition and promote cell shape recovery. Ma R. F.^[56]^ found that PNS reduced the levels of TNF-α, C-reactive protein, and growth differentiation factor-15 by regulating the ATF3/MAP2K3/p38 MAPK and NF-κB signaling pathways and improved cardiac function and fibrosis in rats with myocardial infarction. Shao R. F.^[57]^ proposed that NG-R1 is a unique bioactive component of notoginseng that has anti-inflammatory and antioxidant effects, can reduce the expression of TNF-α, release inflammatory factors in mice with sepsis, and improve myocardial fibrosis. In sum, there are few clinical studies in this field. Future work should focus on strengthening the collection of evidence, developing a standardized system of diagnosis and treatment, and conducting high-caliber multicenter large-sample clinical controlled trials.

1. Cultivation and yield increase research

Nowadays, people are paying greater attention to health, and the demand for TCM is increasing. The cultivation of medicinal plants is closely related to climate, light, soil, water, fertilizer, and seeds, whereas environmental pollution and land loss pose great challenges to the cultivation and production of TCM medicine. Typical keywords in this direction are cultivar breeding, acid soil, genetic diversity, *Fusarium solani*, root rot, heavy metal, seeds, plant cell culture, and salt stress. The research hotspot of notoginseng cultivation centers on improving the polluted environment, eliminating insect pests, and providing a good growing environment for notoginseng. Liu C. L.^[58]^ studied the correlation between total saponins in *P notoginseng* and climate factors, and evaluated the influence of climate factors on the quality of *P Notoginseng*. He found that the lower annual mean temperature and annual temperature difference were conducive to the accumulation of saponin content, which provided a reference for the cultivation and planting of *P notoginseng* in the future. Cun Z.^[59]^ studied photosynth-related pigments, photosynthetic performance, leaf anatomical characteristics, and antioxidant enzyme activity of *P notoginseng* under different light conditions. He believed that the potential cause of the typical shade-tolerant plant *P notoginseng* sensitivity to bright light is PSI photoinhibition and that light damage on the PSII receptor side may cause the inability of the typically shade-tolerant plant to adapt to long-term low-light stress. Li T. T.^[60]^ found that both fennel and garlic essential oils could significantly inhibit notoginseng root rot caused by *Fusarium oxysporum* and help solve the obstacles of notoginseng continuous cropping and other problems with agricultural cultivation. In sum, *P notoginseng*, as a TCM plant, has high medicinal value. It is thus worthwhile to study the rational planting and cultivation of *P notoginseng* to increase the accumulation of effective active ingredients and drug yield.

### 4.2. Strengths and limitations

To our knowledge, this is the first literature assessment and visual analysis of notoginseng and as such provides a reference and guidance for researchers to understand the research situation and hot spots in this field. However, this study has some limitations. First, when excluding the irrelevant literature and combining synonymous keywords, there might have been a subjective understanding bias of the researchers. Second, when we merged the organizations, authors, and keywords, there were some cases in which some content appeared too infrequently to be merged. Third, an author who published 1 paper as the first author was ranked lower than another author who published 2 papers as an intermediate author. However, the first author’s publication was more prestigious and significant than the intermediate author’s. Finally, this article only includes the literature from 3 databases. These 3 databases collect the research results of scholars from all over the world and are universal and representative. This study did not include the literature from databases such as China National Knowledge Infrastructure, Wanfang Database, and VIP Network, which mainly collect Chinese literature. Therefore, there may be some limitations. This paper analyzed only the maps and data generated by the software and might have missed a small amount of research literature. Nevertheless, this visualization-based literature analysis assists in understanding the research trends and directions in the field, laying the foundation for subsequent research.

## 5. Conclusion

We included 1329 articles closely related to the field and used visual analysis software and websites to analyze the annual publishing trends, publishing countries or regions, institutions, authors, journals, keywords, and references in the field. The overall rising trend of publications in the past 24 years is enough to show that the research in this field is gradually increasing, and it is the current research hotspot. Through our analysis, we found that Peoples R China, the United States, South Korea, and Japan were the main research and participating countries. Major research institutions include Kunming University of Science & Technology, the Chinese Academy of Sciences, and the China Academy of Chinese Medical Sciences. The main publications are the *Journal of Ethnopharmacology*, *Molecules*, and *Frontiers in Pharmacology*. Wan Jian-Bo, Wang Dong, and Yang Min were among the authors who published the most papers. At present, the focus of future research in this field consists of 4 aspects, namely, pharmacologic components and quality control research, mechanism of action and pathway target research, applicable disease and therapeutic effects research, and cultivation and yield increase research. In addition, research on micro-standardization, standardization, and objectification of TCM is also developing. We believe that the development of this field will improve in the future.

## Author contributions

**Conceptualization:** Kun Lian, Zhixi Hu.

**Data curation:** Kun Lian, Lichong Meng, Xin Li, Lin Li, Junxian Lei, Ji Ouyang, Yuehang Xu.

**Formal analysis:** Kun Lian, Xin Li, Lin Li, Junxian Lei, Ji Ouyang.

**Investigation:** Kun Lian, Lichong Meng, Lin Li, Zhixi Hu.

**Methodology:** Kun Lian, Lichong Meng.

**Resources:** Kun Lian, Lichong Meng.

**Software:** Kun Lian, Lichong Meng, Xin Li, Junxian Lei, Ji Ouyang.

**Supervision:** Kun Lian, Zhixi Hu.

**Visualization:** Kun Lian, Lichong Meng, Xin Li, Lin Li, Junxian Lei, Ji Ouyang, Yuehang Xu.

**Validation:** Lichong Meng, Xin Li, Zhixi Hu.

**Project administration:** Lin Li.

**Funding acquisition:** Zhixi Hu.

**Writing – original draft:** Kun Lian.

**Writing – review & editing:** Kun Lian.
